# Fecal microbiota impacts development of *Cryptosporidium parvum* in the mouse

**DOI:** 10.1038/s41598-024-56184-1

**Published:** 2024-03-06

**Authors:** Giovanni Widmer, Hannah N. Creasey

**Affiliations:** 1 Cummings School of Veterinary Medicine at Tufts University, 200 Westboro Road, North Grafton, MA 01536 USA; 2 Present Address: Department of Immunology, Harvard Medical School, 77 Avenue Louis Pasteur, Boston, MA 02115 USA

**Keywords:** Microbiome, Infectious diseases

## Abstract

The dependence of *Cryptosporidium* parasites on host cell metabolites suggests that the development of nutritional interventions to limit parasite proliferation should be feasible. Based on this concept, we are testing dietary interventions to affect the enterocytes’ metabolism in a manner that limits intracellular multiplication of the parasite. We hypothesize that changes in the metabolic pathways encoded by the gastro-intestinal tract microbiota may restrict parasite proliferation. To identify taxonomic and metabolic features of the microbiota associated with severity of cryptosporidiosis, as determined by estimating oocyst output, we characterized the fecal microbiota from mice experimentally infected with *Cryptosporidium parvum*. To eliminate the confounding effect of the interaction between co-housed mice, as well as facilitate the identification of microbiota markers associated with severity of cryptosporidiosis, fecal microbiota from individually caged mice were analyzed. Variation partitioning analysis applied to 16S sequence data from 25 mice belonging to four experiments shows that experiment was by far the biggest source of microbiota variation. Severity of cryptosporidiosis explained a smaller, though significant, fraction of microbiota variation. Notably, this effect was significant in the pre-patent phase of the infection, before mice excreted oocysts. These results are consistent with the pre-patent intestinal microbiota having a modest, but measurable, effect on cryptosporidiosis.

## Introduction

The lack of effective treatments and vaccines against cryptosporidiosis is motivating our search for alternative interventions to reduce the impact of this infection in vulnerable populations. The multiplication of *Cryptosporidium* inside intestinal epithelial cells, a phase of the life cycle known as “merogony”, and the metabolic dependence of the parasite on the host cell for essential metabolites^1,2^, imply that parasite multiplication can potentially be modulated by altering the host cell metabolism. The impact of diet on the enterocyte’s metabolism has been the focus of research for years^3^. For instance, deprivation of dietary fiber reduces the intestinal concentration of butyrate, the main source of energy for enterocytes, and negatively affects the integrity of the mucus layer^4^. An in vitro study consistent with this model found that *C. parvum* growth in cultured adenocarcinoma cells was inhibited by short chain fatty acids added to the culture medium^5^. The feasibility of impacting the enterocytes’ metabolism using nutritional intervention is consistent with this knowledge and with the role that the microbiota’s metabolites play in regulating the enterocytes’ metabolism and secretion of protective mucus^6,7^. The metabolic flexibility of enterocytes^8^ also supports the feasibility of this approach. Although much research has focused on elucidating host-microbiota interaction in the healthy and infected GI tract^9–12^, the feasibility of harnessing this knowledge to prevent the most severe consequences of intracellular enteric pathogens has to our knowledge not been extensively explored. The interdependence between enterocyte and microbiota motivates our search^13–15^ for nutritional interventions capable of impacting *C. parvum* proliferation as low-cost alternatives to future vaccines and drugs.

To study the interaction between host and *Cryptosporidium* parasites, immunosuppressed mice are commonly used^16,17^. As with any research model, translating insights gained from murine models to the clinic has significant limitations. Primarily, the mouse is not a natural host of *C. parvum*, requiring immunosuppression or undernutrition for the infection to take hold and the parasite to proliferate. This requirement excludes studying the role of acquired immunity in the interaction between host, intestinal microbiota and pathogen. On an anatomical level, in contrast to the mouse, the human cecum is small and does not ferment, a difference particularly relevant to the study of the impact of the intestinal microbiota in regulating infection. An emerging and less explored drawback of modelling the human microbiota in the mouse is the unexplained difference in the taxonomy of the native intestinal microbiota in different mouse strains and different lots of mice. 16S amplicon sequencing has begun to uncover vastly different intestinal microbiota in mice used in different studies, even when mice originated from the same vendor^15,18,19^. This unintended variability has the potential to negatively affect reproducibility, particularly given the large number of physiological and behavioral phenotypes impacted by the gut ecology.

To our knowledge, molecular mechanisms linking *Cryptosporidium* proliferation with the GI microbiota have not been elucidated. With the goal of identifying microbiota markers associated with the course of cryptosporidiosis, here we report a meta-analysis of four independent experiments comprising 25 individually caged mice experimentally infected with *C. parvum*. A part of the data were included in a previous publication describing the effect of caging on the development of *C. parvum*. Here we show that 16S amplicon sequencing of pre-patent fecal microbiota revealed a modest impact of the intestinal microbiota on the severity of cryptosporidiosis. The magnitude of this effect was smaller than the taxonomic and functional β diversity between microbiota populating mice from different lots. After subtracting the effect of the infection and mouse lots, a relatively large proportion of microbiota variability remained unexplained. Gaining a better understanding of the source of this variability would greatly improve reproducibility, not only of the murine model of cryptosporidiosis, but also of other enteric conditions.

## Results

### Aggregate analysis of 16S sequence data from four experiments

Because of the potential for caging conditions to affect the course of cryptosporidiosis^15,20^, in this study we included only individually caged animals. The goals of these analyses were twofold; the first set of analyses focused on the entire dataset of 108 samples collected between day − 3 post-infection and the end of each experiment. The scope of the second set of analyses was to evaluate whether an impact of the gut microbiota on the course of cryptosporidiosis could be detected. Because of the reported effect of cryptosporidiosis on the intestinal microbiota, these analyses included only 48 samples collected before oocysts were observed in the feces.

**Figure 1:**
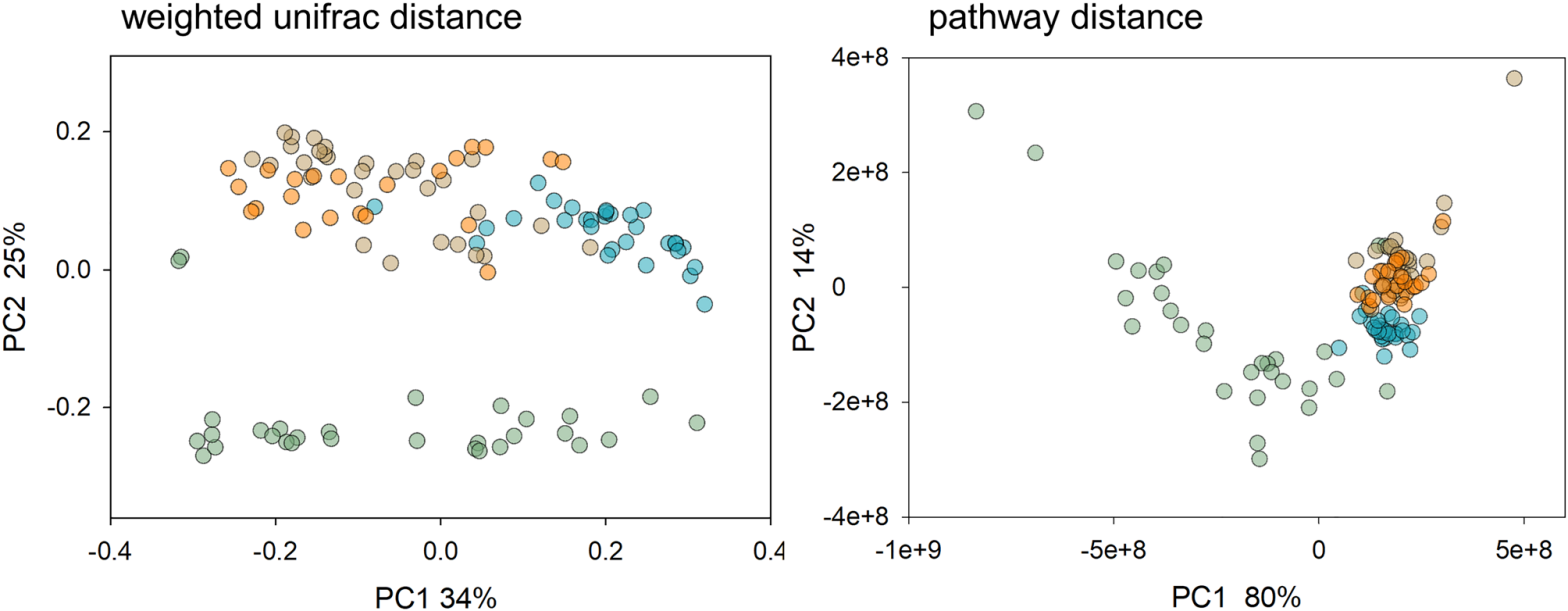
Principal Coordinate Analysis of 108 fecal microbiota of individually caged mice which were part of four independent experiments. Turquoise, experiment 35; green, experiment 37; beige, experiment 38; orange, experiment 40. Left, PCoA of 16S sequence data based on weighted UniFrac distance; right, 16S sequences were translated into metabolic pathways using PICRUSt2 and pairwise distances between pathway abundance values visualized by PCoA. Percent variation explained by each axis is indicated.

A PCoA of 16S sequences from 108 fecal samples originating from four distinct experiments and a total of 25 individually caged mice (Table 1) revealed significant clustering of the fecal microbiota according to experiment. In particular, experiment 37 microbiota clearly clustered separately from the microbiota of the three other experiments (experiments 35, 38, 40) (Fig. 1). Clustering was not only apparent in 16S sequence data, but also when inferred abundances of metabolic pathways were analyzed. As expected from the PCoA plots, ANOSIM^21^ confirmed the statistical significance of the experiment on sample clustering based on 16S sequences (R = 0.64, *p* < 2e−5; Table 2). We used constrained ordination methods to quantify the effect of two independent variables on the observed microbiota profile variation at the OTU and metabolic function level. These analyses were intended to quantify the proportion of OTU and metabolic pathway variation attributed to mouse lot and to the intensity of the infection (Fig. S1, Table 3, Table S1). As expected from the PCoA (Fig. 1), mouse lot (i.e., the experiment) explained a relatively large percentage of OTU variation (29.0%, pseudo-F = 14.1, *p* ≤ 0.001, n = 108). To subtract the effect of the experiment, this variable was defined as covariate, while oocyst output was defined as the independent variable. This analysis shows that the severity of the infection had a significant effect on the microbiota (pseudo-F = 3.2, *p* = 0.004, n = 108). Oocyst output explained only about one tenth of the microbiota variation compared to experiment (3.0% vs. 29.0%). Inspection of the PCoA plot (Fig. 1) and the R values in Table 2 do not reveal an effect of mouse strain nor mouse gender. The weighted UniFrac distance between samples from experiment 40 (the only one of the four experiments which did not use CD-1 mice) and the other experiments does not exceed the distance among the three other experiments which used CD-1 mice. Also noteworthy is the fact that outbred mice did not harbor a more variable microbiota (experiment 35, mean weighted UniFrac = 0.231; experiment 37, mean weighted UniFrac = 0.311; experiment 38, mean weighted UniFrac 0.255; experiment 40, mean weighted UniFrac = 0.345).

**Table 1:** Summary of experiments.

Experiment^1^	Mouse strain	Sex	Number mice	Number samples	*C. parvum* isolate	Date of infection^3^
35	CD-1	Female	7	30	MD	07/15/20
37	CD-1	Female	6	30	TU114	02/23/21
38	CD-1	Male	6	30	TU114	03/25/21
40	B6J.C3-Sst1^2^	Male	6	22	TU114	02/21/22

^1^Parts of experiment 35–38 data were the subject of previously published analyses^15^.

^2^See^49^.

^3^MM/DD/YY.

**Table 2:** Microbiota 16S sequence data clustering by mouse lot^1^.

Comparison	R	*p*
35–37–38–40^2^	0.64	< 2e−5
35–37	0.76	< 2e−5
35–38	0.67	< 2e−5
35–40	0.77	< 2e−5
37–38	0.72	< 2e−5
37–40	0.61	< 2e−5
38–40	0.27	< 2e−5

^1^Each experiment used a different lot of mice.

^2^See Table 1 for details of experiments 35, 37, 38 and 40.

**Table 3:** Results of constrained ordination analyses.

Dependent variables	Independent variable	Pseudo-F^1^	*p*	% variation explained	n^2^	Comment
OTU	Mean oocyst	3.2	0.004	3.0	108	All days
	Mean oocyst	1.1	n.s		48	Pre-patent^3^
PWY	Mean oocyst	7.9	0.001	7.1	108	All days
	Mean oocyst	3.1	0.012	3.1	48	Pre-patent

^1^Experiment defined as covariate.

^2^Number of samples.

^3^Samples collect between day − 3 to + 3 post-infection, i.e., from 3 days pre-infection to 3 days post-infection.

A second set of analyses focused on pre-patent samples (n = 48) only. The goal was to estimate the effect of the microbiota metabolic function and mouse lot on oocyst output. Variation Partitioning Analysis^22^ showed that the conditional effect, i.e., the unique effect of mouse lot on pathway abundance represented 83% of the explained variation, or roughly half (56.3%) of the total variation. In contrast, oocyst output represented 2.4% of the explained metabolic pathway variation or 1.6% of total variation. Consistent with the results summarized in Table 3, the effect of both independent variables (experiment and oocyst output) was statistically significant (pseudo-F = 27.4, *p* = 0.001 and pseudo-F = 3.1, *p* = 0.012, respectively, n = 48). In this analysis, 32.3% of variation is not explained by these variables or their interaction. Running the analogous variation partitioning analysis using Operational Taxonomic Unit (OTU) abundance as dependent variables, instead of pathway abundance, revealed a slightly smaller proportion of variation explained by the experiment and oocysts output (48.9%, *p* = 0.001). With 0.2% (n.s.) of total variation explained (conditional effect), oocyst output also explained less OTU variation than pathway variation. Based on the analysis of OTU abundance, 49.6% variation remained unexplained.

To investigate the bacterial taxonomy underlying the clustering of microbiota from different mouse lots, Canonical Correspondence Analysis (CCA) based on the samples’ OTUs as dependent variables and experiment as the sole independent variable was applied. To exclude the effect of cryptosporidiosis on the microbiota, this analysis included only 48 samples collected during the pre-patent phase of each experiment, i.e., from day − 3 post-infection until day + 3 post-infection. The taxonomy underlying the clustering according to experiment was examined by classifying the OTUs which are most closely associated with this variable. This association was quantified using the FitE variable^23^. Among a total of 361 OTUs in this dataset, for 17 OTUs the percent fit with experiment exceeded 90%. These OTUs belong primarily to the family Muribaculaceae (14 OTUs). The remaining 3 OTUs belong to the Bacteroidaceae (1 OTU), Ruminococcaceae (1 OTU) and Clostridiaceae (1 OTU). In contrast, the OTUs which are least associated with the independent variable (< 10% percent fit, n = 6) were more diverse (Clostridiales, 2 OTUs, Peptostreptococcaceae, 1 OTU; Erysipelotrichaceae, 1 OTU; Ruminococcaceae, 1 OTU; Lachnospiraceae, 1 OTU). The relative abundances of two OTUs in the best-fitting group (OTU006 and OTU008) and, as comparison one OTU with a very low fit of 11%, (OTU005) are illustrated in Fig. S1.

### Severity of infection and the fecal microbiota; cause or effect?

**Figure 2:**
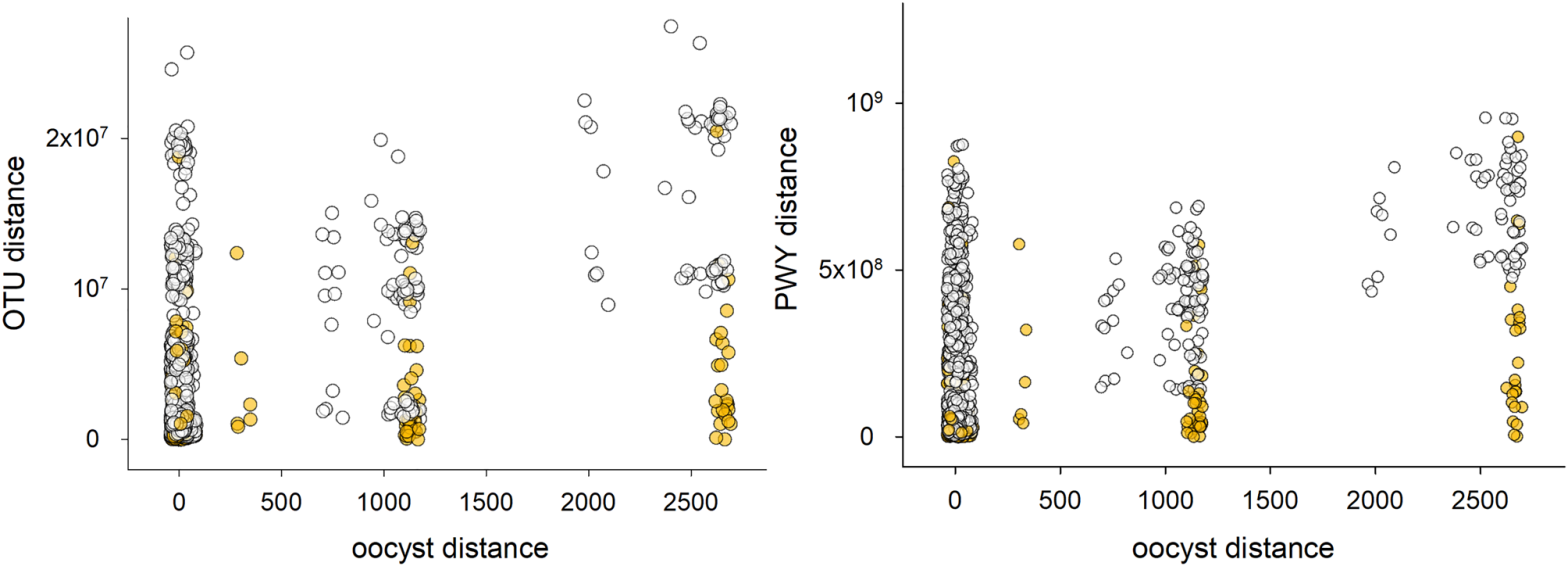
Association of pre-patent microbiota and the course of cryptosporidiosis. Taxonomic β diversity (left) and functional β diversity (right) are positively correlated with the difference in the course of the infection. Left, taxonomic distance vs. oocyst distance (r = 0.42, n = 1128, *p* = 6.3e−50). Right, pathway distance vs. oocyst distance (r = 0.44, n = 1128, *p* = 2.8e−54). Oocyst distance is defined as the square of the difference between mean oocyst output of two mice. The analysis is based on 48 pre-patent samples. Samples collected after day 3 post-infection are excluded. Yellow symbols indicate comparisons between samples from the same experiment.

To further assess whether the intestinal microbiota impacts the course of cryptosporidiosis, the correlation of oocyst distances vs. OTU distances (see Methods) and oocyst distances vs. pathways distances was analyzed. To distinguish between cause and effect, i.e., the microbiota impacting the course of the infection vs. the infection impacting the microbiota, the correlation analysis focused on 48 samples collected during the pre-patent phase of each experiment, before any oocysts were detected in the feces. Consistent with the view that *Cryptosporidium* proliferation is impacted by the pre-patent microbiota, a significant correlation between oocysts distances and microbiota distance was observed (Fig. 2). The pre-patent period is defined as days − 3 to + 3 post-infection, i.e., from 3 days before the mice were infected until 3 days after infection. This time period was selected to maximize the number of samples while excluding samples collected when the mice were excreting oocysts in the feces. Although the microbiota taxonomy explains a very small and statistically not significant portion of OTU variation, RDA based on the inferred abundance of metabolic pathways returned a significant association between severity of infection and the metabolic function prior to the mice developing a patent infection (Table 3). This result indicates a positive correlation between microbiota β diversity and difference in the severity of the infection (Fig. 2). In other words, mice harboring functionally more divergent microbiota also experienced infections of different severity. Because the correlation analysis focused on pre-patent microbiota only, these results are indicative of the microbiota impacting the proliferation of *C. parvum* as opposed to the microbiota responding to the infection^24,25^.

### Focus on experiment 37

**Figure 3:**
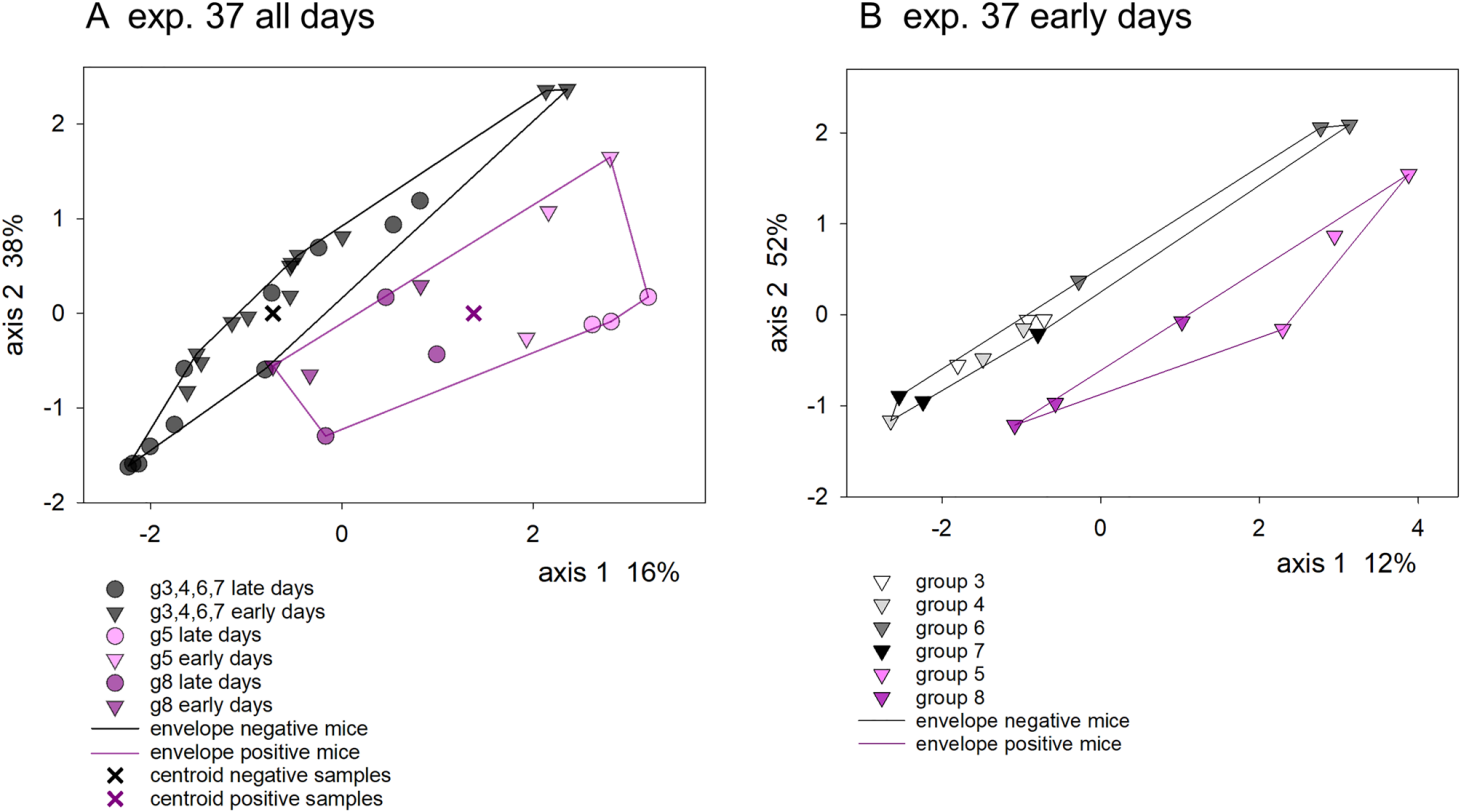
Redundancy analysis biplots showing the sample scores in relation to the sole independent variable represented by mean oocyst output. The distance between sample symbols (circles and triangles) approximates the β diversity calculated from 223 normalized metabolic pathways abundance values. All samples originate from experiment 37, (**A**) all samples; (**B**) pre-patent samples. Purple and black polygons enclose samples of mice that became infected and shed oocysts (mouse g5 and g8) and mice that did not (mouse g3, g4, g6, g7), respectively (Fig. S2). Pre-patent samples are represented with triangles and samples from the remaining days are indicated with circles. Left, all samples, n = 35. Axis 1 points in the direction of steepest increase in mean oocyst count in the ordination plane and explains 16% of the metabolic repertoire variability. Right, RDA biplot of pre-patent samples only (n = 18) shows a clear separation between mice that became positive and excreted oocysts (purple) and those that did not (black, grey, white). Axis labels show percent explained variation.

The variability in the intensity of cryptosporidiosis among mice was particularly apparent in experiment 37 (Fig. S2). Two of six mice developed a severe infection, whereas four mice did not excrete any oocysts. This observation was exploited to search for markers of severe infection in experiment 37 microbiota. OTU or metabolic pathway relative abundance values were input as dependent variables into separate RDAs using oocyst output as the explanatory variable. A biplot based on 35 samples collected during the entire experiment (Fig. 3A) revealed a clear separation of the microbiota’s metabolic repertoire between the mice that did not experience a measurable infection and the two mice which became heavily infected. RDAs showed that OTU relative abundance and metabolic pathways were both significantly associated with infection (pseudo-F 10.1, *p* = 0.0001 n = 30; pseudo-F = 5.8, *p* = 0.0007, n = 30, respectively). Oocyst output explained a relatively large proportion of OTU variation (26.5%) and pathway abundance (14.2%). As expected, by focusing on a single experiment the intensity of the infection explained a much larger proportion of microbiota variation than in the aggregated analysis with four experiments described above (Table 3). Because of our interest in microbiota markers which could predict the severity of cryptosporidiosis, experiment 37 samples excreted on day − 1, + 1 and + 3 post-infection (n = 18) designated herein as “pre-patent”, were analyzed separately. This approach is intended to exclude the secondary effect that *C. parvum* proliferation is known to exert on the fecal microbiota^24,26^. Figure 3B shows a RDA biplot of 18 pre-patent samples revealing a clear separation of microbiota metabolic pathways from mice that shed oocysts and those that did not. Given the relatively large effect of individual mice on the microbiota apparent in Fig. 3A and B, to test the statistical significance of the difference between microbiota function in infected and uninfected mice, the effect of the individual mouse was subtracted by defining mouse as a covariate, while oocyst output served as the sole independent variable. Despite the small sample size, this analysis showed that oocyst output was significantly associated with pre-patent microbiota function (pseudo-F = 3.3, *p* = 0.03). As expected from the large intra-group variation apparent in Fig. 3B, linear discriminant analysis identified relatively few metabolic functions significantly associated with the course of the infection. Adenosine nucleotides degradation II, colanic acid building blocks biosynthesis, superpathway of GDP-mannose-derived O-antigen building blocks biosynthesis and superpathway of UDP-glucose-derived O-antigen building blocks biosynthesis were significantly more abundant in the microbiota of uninfected mice (Table S2) and can thus be viewed as having a protective effect. No pathways were overrepresented in pre-patent microbiota from mice that did become infected. The negative association of the above mentioned four metabolic pathways with experiment 37 susceptibility to *C. parvum* infection was also identified with RDA (Table S2).

## Discussion

We analyzed the course of cryptosporidiosis in four independent cohorts of individually caged mice focusing on the association of two variables, experiment and severity of *C. parvum* infection, with the taxonomy and inferred metabolic function of the fecal microbiota. The analysis of fecal microbiota collected before the mice excreted oocysts enabled us to test the extent to which the microbiota impacts the course of cryptosporidiosis, i.e., before the microbiota is affected by the infection. Although infection explained a smaller proportion of microbiota variability compared to the lot of mice, this observation justifies the search for microbiota markers linking parasite proliferation and fecal microbiota, and is relevant for developing alternative treatments for cryptosporidiosis.

The analysis of the variability of the fecal microbiota revealed that between 32 and 49% of pathway and OTU abundance, respectively, are not explained by the mouse lot and oocyst output. This observation raises interesting questions regarding the source of the relatively large proportion of unexplained variation. The quality controls included in the laboratory procedures and in the sequence analysis pipeline described in Methods show that technical variation had a minor effect. The main limitations of 16S amplicon sequencing is that the method outputs relative abundance values and that it does not distinguish between species. Absolute abundances and species-level resolution could possibly uncover microbiota markers with more explanatory power. Unexplained variation may also originate from the method used to quantify the concentration of fecal oocysts. Like all oocyst enumeration methods, microscopic analysis of fecal smears introduces errors which are expected to contribute to an underestimate of the proportion of microbiota variability associated with oocyst output. Oocyst output is potentially intermittent^27^ which may have led to over- or underestimating average oocyst output. Also to be considered is the presence of other biological factors impacting the intestinal microbiota, like inter-mouse variation caused by random drift of bacterial communities in the gut or the amount of dexamethasone ingested with the drinking water. Supporting this view is the mouse-to-mouse variability apparent in the analysis shown in Fig. 3. In contrast, genetic background and inbred vs. outbred breeding history do not seem to have impacted the results, as demonstrated by the analysis presented in Fig. 1. In fact, the microbiota of B6J.C3-Sst1 mice used in experiment 40 clustered relatively closely with those from experiment 35 and 38, whereas CD-1 microbiota in experiment 37 mice clustered separately from microbiota originating from the other two experiments performed with CD-1 mice. Identifying additional factors explaining microbiota variability in addition to those examined here would increase the statistical power of future research aimed at better understanding the interaction between host, parasite and microbiota. Also worthy of additional scrutiny is the animal model, in particular the question whether mice immunosuppressed with dexamethasone are relevant models of human cryptosporidiosis. Two alternatives worthy of consideration are protein-malnourished mice fed a 2% protein diet^28,29^ and immunocompetent mice infected with *C. tyzzeri*^30^.

With the observed large differences in microbiota profile among different mouse lots, it is conceivable that mechanisms modulating the development of *C. parvum* could vary according to the lot, strain and breeding facility. Identifying such mechanisms will require a consistent and reproducible intestinal microbiota. Depleting the native microbiota with antibiotics followed by inoculation with a standardized bacterial population^31^ may minimize microbiota variability among individual experiments. The observed fluctuation in microbiota composition, even among mice originating from the same vendor, raises research reproducibility issues as has previously been discussed^32,33^.

The extracellular stages in the *Cryptosporidium* life cycle (sporozoites, meronts, microgametes and oocysts) do not replicate. Our working hypothesis predicts that the interaction between the parasite and the microbiota takes place during the replicative phase (merogony), because the dividing parasite depends on host cell metabolites^34,35^. In contrast, the metabolism of a non-proliferating stage is presumed to be limited to functions required for viability and perhaps motility in the case of the sporozoites and the microgametes. Dietary measures to mitigate the severity of cryptosporidiosis should therefore focus on the metabolism of the host cell. Such interventions could act on enterocytes but also on goblet cells to promote mucin secretion. The goal of the experiments described here and published previously^15^ aim to identify molecular mechanisms explaining the interaction between microbiota metabolism and the course of cryptosporidiosis. To distinguish between cause and effect, microbiota collected no later than 3 days post-infection were analyzed separately from samples collected later in the experiment. This approach excludes that any association between microbiota profile and severity of infection could be caused by the effect of the infection on the intestinal ecosystem previously demonstrated^24–26,36^. It is tempting to speculate that the metabolic pathways identified by linear discriminant analysis of experiment 37 pre-patent samples (adenosine nucleotides degradation and the three biosynthesis pathways over-represented in the four mice that did not develop an infection) are somehow protective. Although the LDA analysis and RDA results are in agreement, concluding on the general the validity of this result is clearly premature. To identify microbial metabolites exerting an effect on intracellular parasite multiplication, metabolomics analyses together with the analysis of the intestinal metagenome will be needed. In light of the extensive β diversity between microbiota in different lots of mice, metabolic functions identified in multiple experiments using different lots of mice are worthy of further investigation. The Adenosine Nucleotides Degradation pathway is the only catabolic pathway among the 4 pathways identified by linear discriminant analysis. According to the published literature, no connection between this pathway and enteric infections has to our knowledge been reported. According to the same linear discriminant analysis, the colanic acid building blocks biosynthesis pathway was overrepresented in the microbiota of 37 mice which did not develop an infection. The literature linking colanic acid and infection primarily refers to the synthesis and regulation of colanic acid in *Escherichia coli*. This molecule is found on *Enterobacteriaceae* exopolysaccharides^37^ but hypothesizing on the link between upregulation of this pathway and increased resistance to *C. parvum* would be speculative. The same conclusion is reached when searching the literature for evidence linking the two remaining biosynthetic superpathways, GDP-mannose-derived O-antigen building blocks biosynthesis and UDP-glucose-derived O-antigen building blocks biosynthesis.

In conclusion, investigating the impact of the intestinal microbiota on *C. parvum* development in the GI tract and identifying underlying mechanisms is of scientific and clinical interest. Because of the impact of the infection on the intestinal microbiota^24^, discriminating between the effect of the infection on the microbiota and the more important opposite effect was enabled by separately analyzing fecal microbiota collected before oocysts were present in the feces. The detection, despite the small sample size, of a modest, but significant, association of predicted metabolic function of the fecal microbiota with fecal oocyst concentration is consistent with our hypothesis that dietary interventions aimed at reducing parasite proliferation and minimizing symptoms should be explored. The fact that a significant microbiota effect was detected despite the variation in the fecal microbiota profile and the relatively large proportion of unexplained microbiota variability strengthens our hypothesis.

## Methods

### Mouse experiments and oocyst quantification

All methods were carried out in accordance with relevant guidelines and regulations. The study was carried out in compliance with the ARRIVE guidelines. Animal experiments were approved by the Tufts University Institutional Care and Use Committee under protocol G2021-115.

In a previous publication focused on the effect of caging on the course of cryptosporidiosis^15^, samples from three experiments (Table 1, #35, #37, #38) with group caged and individually caged mice were analyzed to explore the effect of caging. All samples analyzed in the present contribution originated from mice caged individually, including those from experiments 35, 37 and 38 described previously^15^. CD-1 mice between four and six weeks of age were purchased from Charles River Laboratories (Wilmington, MA, USA). CD-1 mice were used in experiments 35, 37 and 38. The mice used in each experiment were ordered separately (Table 1) and came from a different breeding facility. All mice included in a given experiment were purchased together and presumably originated from the same facility. None of the experiments overlapped in time. Experiment 40 used inbred B6J.C3-Sst1 mice (Jackson Laboratories, Bar Harbor, Maine, USA) bred in-house (Table 1). Mice were housed individually in sterile filter-top cages approximately 27 × 17 × 20 cm in size. The cages contained bedding of sterile corn chips and were maintained at a temperature of 20 °C in a 12-h light–dark cycle. On three to four occasions during each experiment coinciding with the peak oocyst shedding period, mice were transferred overnight individually to wire-bottom cages to facilitate the collection of fecal material. Mice were treated as much as possible in an identical manner across all four experiments and were fed the same diet. Starting on the day of arrival in the facility (or removal from the breeding colony for experiment 40), sterile drinking water supplemented with 16 mg/l dexamethasone 21-phosphate disodium (Thermo Fisher Scientific, Waltham, Massachusetts, USA) was provided ad libitum^16^. Mice had unrestricted access to standard sterile rodent chow containing 18% protein (Teklad 2018, Envigo, Indianapolis, IN, USA). Oocyst were purified from fecal material on step gradients of 10% w/v—25% w/v Nycodenz in water as described^38^. Mice were orally infected with 50,000 oocysts of isolate MD^39^ or TU114^40^ suspended in sterile water. Oocysts shedding in the feces was quantified by microscopically counting acid-fast stained oocysts in fecal smears^41^ by a blinded observer. Ten replicate counts were acquired at 400 × magnification as described^15^. Mean oocyst concentration was estimated for each mouse by averaging the oocyst counts over the number of observations. Oocyst distance is defined as the square of the difference between two mean oocyst concentration values. A total of 112 fecal samples from 25 mice belonging to four separate experiments were included in the analyses presented here. 16S sequence data from experiments 35, 37 and 38 were included in a previous publication^15^. Analyses combining 16S sequences and corresponding metadata, such as experiment and mean oocyst shedding, comprise 108 samples. The difference in sample number is due to the exclusion of two experiment 40 samples collected from mice which were euthanized on day 7 post-infection. Two pairs of replicated experiment 40 samples were pooled, leaving a total of 108 samples.

### 16S amplicon sequencing and bioinformatics

V1V2 amplicons were amplified from the bacterial 16S ribosomal RNA gene using primers 27F and 338R^42^. PCR and sequencing procedures were previously described^13^. Sequence denoising and analysis were performed in *mothur*^43^. The outputs of this analysis were primarily taxonomic classifications and sample x variable data matrices designated “shared” files in *mothur*. OTUs were formed with a 97% similarity cut-off. The significance cut-off for taxonomic classification was set at 70%. Linear Discriminant Analysis was performed using program LefSe^44^ as implemented in *mothur*.

The following distance measures were used: (1) Weighted UniFrac distance^45^ was used to quantify β diversity between samples based on curated 16S sequences. (2) The difference in the severity of the infection between two mice was calculated as squared difference between mean oocysts output, where mean oocyst output is equal to the sum of oocysts counts divided by the number of counts. (3) The distance between samples based on OTU abundance and metabolic pathway abundance is equal to the squared difference between abundance values for each OTU and pathway, respectively, summed over all variables. Herein, these distance values are referred to as “OTU distance” and “Pathway Distance”. Weighted UniFrac distance^45^ was calculated in *mothur*^43^. GenAlEx^46^ was used to calculate Principal Coordinate Analysis sample scores, i.e., the sample coordinates on the principal axes.

Bacterial metabolic pathway abundances were predicted from 16S data using PICRUSt2^47^. Sample x Amplicon Sequence Variants (ASVs) matrices for inputting into PICRUSt2 were generated with command *make.shared* in *mothur* with the argument label = ASV.

The association between independent variables, like mouse lot or mean oocyst output, and the dependent variables (OTUs or metabolic pathway) was tested using constrained ordination. Depending on the distribution of the dependent variables, RDA or CCA^48^ was applied. Ordination analyses were performed in CANOCO v.5^23^ and in GenAlEx v.6.5^46^. Linear Discriminant Analysis (LDA) was performed with program LefSe^44^ as implemented in *mothur*. Variation Partitioning analysis was performed in CANOCO.

### Quality control

As 16S amplicons from different experiments were sequenced in different libraries, we evaluated the effect of the sequencing reaction on the sequence data. To this aim, a separate V1V2 amplicon amplified from a synthetic bacterial population (BEI Resources HM-782D) was included in each library. Sequences from these amplicons were compared using the same *mothur* pipeline and weighted UniFrac distance between all pairwise combinations were calculated (Table S3). This analysis shows no evidence of the library or the sequencing reaction having impacted the results. On average, pairwise distances between there controls were very small, averaging 0.04 UniFrac distance units (6 comparisons, SD = 0.008). A second QC procedure to assess the quality of the sequence data, and to estimate technical variation, was the inclusion in each sequencing library of uniquely barcoded amplicons originating from the same fecal sample. The weighted UniFrac distance between duplicated amplicons was 0.151, 0.156, 0.174 (experiment 35; 3 duplicated samples), 0.039 (experiment 38, one duplicated sample), 0.043 and 0.036 (experiment 40, 2 duplicated samples).

### Supplementary Information

Supplementary Information.

## Data Availability

16S sequence data were deposited in FASTQ format in the Sequence Read Archive, National Library of Medicine, National Center for Biotechnology Information, under project numbers PRJNA838969 and PRJNA841949.
