# Supplementary material for: Fecal microbiota impacts development of Cryptosporidium parvum in the mouse
Source: Sci Rep. 2024 Mar 6;14:5498. doi: 10.1038/s41598-024-56184-1 (PMC10917813; doi:10.1038/s41598-024-56184-1)
Supplement: Supplementary file 1 — Supplementary Information. [file 41598_2024_56184_MOESM1_ESM.pdf]

Fig. S1. Mean oocysts output by mouse and experiment

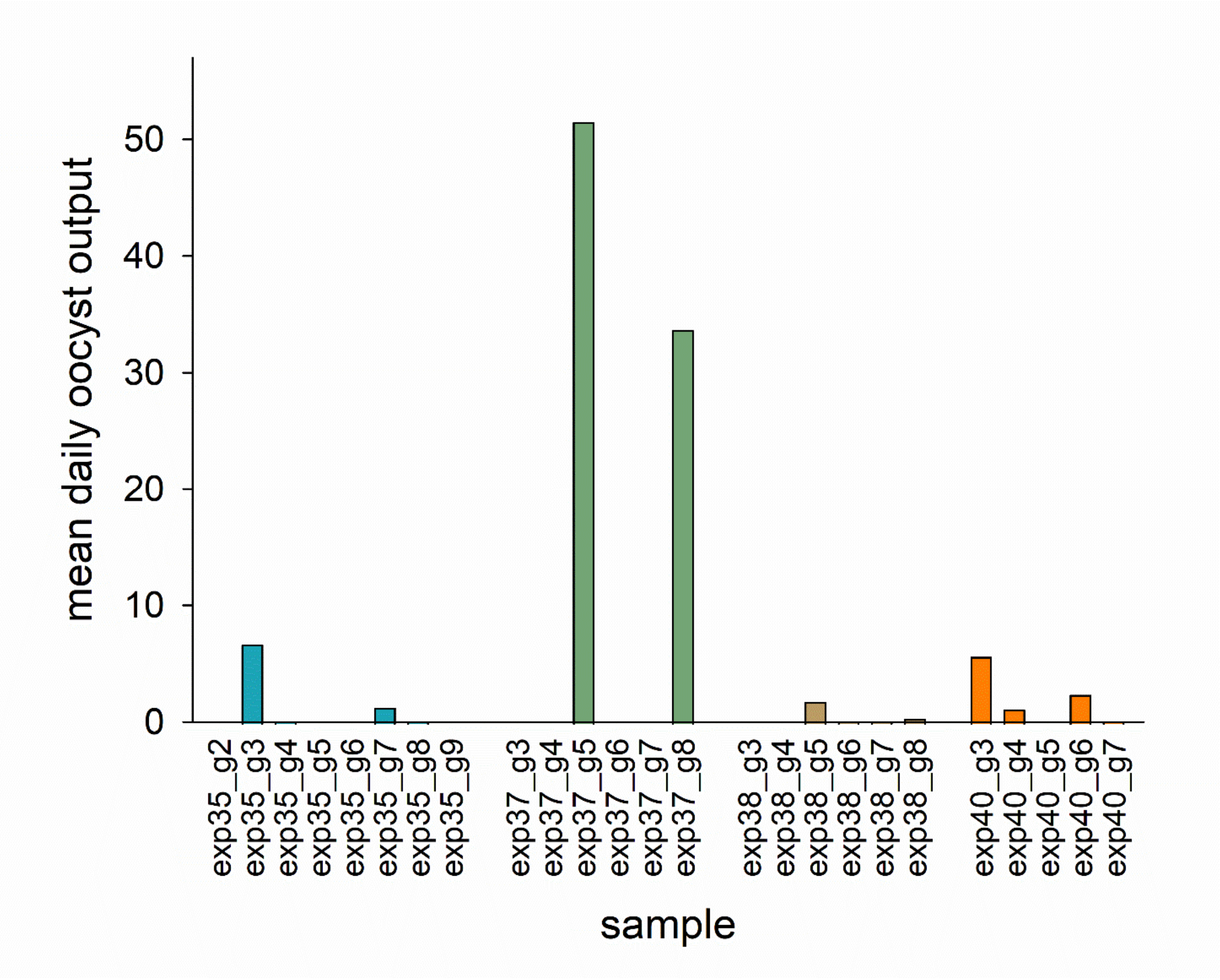

Fig. S2. The relative abundance of OTU006 and OTU008 closely correlates (>90% fit) with experiment. OTU005 is shown as example of an OTU that is not associated with experiment (11% fit). Each bar represents a sample. Colors represent experiment; Turquoise, experiment 35; green, experiment 37; beige, experiment 38; orange, experiment 40. For clarity, samples with zero OTU abundance are represented with a bar equal 0.5%. Relative abundance values of each OTU across the four experiments sum to 100%.

The classification of the OTUs is as follows:
OTU006:Firmicutes;Clostridia;Clostridiales;Clostridiaceae_1; Candidatus_Arthromitus.
OTU008: Bacteroidetes; Bacteroidia; Bacteroidales; Muribaculaceae; Muribaculaceae_ge.
OTU005: Firmicutes; Erysipelotrichia; Erysipelotrichales; Erysipelotrichaceae; Turicibacter(100).

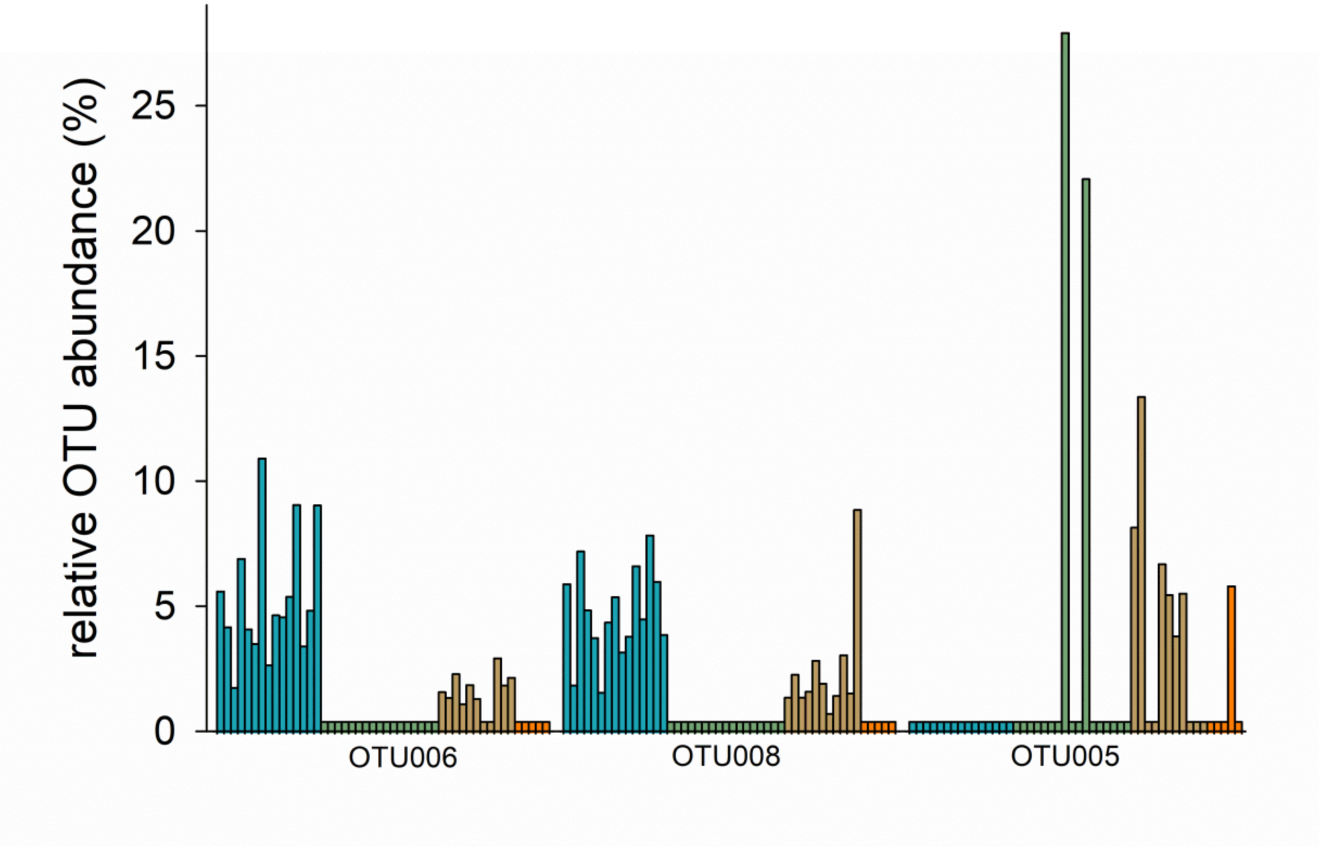

Explanatory notes for Table S1.

	The numbers are raw oocyst counts in ten 400x microscope fields.
Column A	Date (DDMMYY); example: Day of infection 250321 = March 25, 2021
Column B	"day PI" indicates day post-infection where day of infection = day 0
Row 2	mouse ID
mean	total count / number of counts

Table S1. Experiment 35

		mouse							
date (DDMMYY)	day PI	**g2**	**g3**	**g4**	**g5**	**g6**	**g7**	**g8**	**g9**
210720	6	0	4	1		0	0	1	0
240720	9	0	7	0	0	0	0	0	0
270720	12	0	3	0	0	0	0	0	0
290720	14	0	3	0	0	0	0	0	0
080320	19	0	17	0	0	0	7	0	0
mean		0.0	6.8	0.2	0.0	0.0	1.4	0.2	0.0

Table S1. Experiment 37

		mouse					
date (DDMMYY)	day PI	**g3**	**g4**	**g5**	**g6**	**g7**	**g8**
010321	6			14			1
020321	7	0	0	32	0	0	0
030321	8	0	0	73	0	0	28
040321	9	0	0	26	0	0	78
050322	10	0	0	68	0	0	21
080321	14	0	0		0	0	23
090321	15	0	0	19	0	0	5
110321	17	0	0	85	0	0	114
150321	21			64			
160321	22			83			
mean		0.0	0.0	51.6	0.0	0.0	33.8

Table S1. Experiment 38

date (DDMMYY)	day PI	mouse<br>**g3**	**g4**	**g5**	**g6**	**g7**	**g8**
310321	6	0	0	0	0	0	0
010421	7	0	0	0	0	0	0
020421	8	0	0	0	0	0	0
050421	11	0	0	2	0	0	0
060421	12	0	0	0	0	0	0
070421	13	0	0	0	1	0	0
080421	14	0	0	4	0	2	3
090421	15	0	0	8	1		
120421	18	0	0	0	0	0	1
130421	19	0	0	5	0	0	0
mean		0.0	0.0	1.9	0.2	0.2	0.4

Table S1. Experiment 40

		mouse					
date (DDMMYY)	day PI	**g3**	**g4**	**g5**	**g6**	**g7**	**g8**
280222	7	0	0	0	0	0	5
010322	8	23	0	0	0	0	
030322	10	0	0	0	7	1	
070322	14	0	5	0	3	0	
mean		5.8	1.3	0.0	2.5	0.3	5.0
							excluded

Table S1. Results of Lefse and RDA analysis of metabolic pathways in experiment 37 early day samples.

LEFSE

Pathway	Infection	LDA	p-value
PWY-7328	negative	2.053	0.004
SALVADEH	negative	2.275	0.031
PWY-7323	negative	2.488	0.039
COLANSYN	negative	2.625	0.031

RDA

Pathway[1]	Pathway RDA score[2]
P163-PWY	-0.8547
PWY-7328	-0.7869
HISDEG-PWY	-0.7858
PWY-7003	-0.6919
PWY-6588	-0.68
GALACTUROCAT-PWY	-0.6657
PWY-7242	-0.659
GALACT-GLUCUROCAT-PWY	-0.6566
PWY-7315	-0.6492
GLUCUROCAT-PWY	-0.6491
PWY-6507	-0.6225
PWY-6478	-0.5939
PYRIDNUCSYN-PWY	-0.5779
SALVADEHYPOX-PWY	-0.565
P562-PWY	-0.5622
PWY-5154	-0.5529
PWY-7237	-0.55
TRPSYN-PWY	-0.5385
PWY-6608	-0.5363
P461-PWY	-0.5324
PWY-5189	-0.5318
PWY-5509	-0.5237
PWY-6269	-0.5234
COBALSYN-PWY	-0.5233
PWY-6545	-0.5218
P108-PWY	-0.5184
PWY-5188	-0.5184
PWY-6703	-0.5131

DESCRIPTION

Code	MetaCyc description
PWY-7328	superpathway of UDP-glucose-derived O-antigen building blocks biosynthesis
SALVADEHYPOX-PWY	adenosine nucleotides degradation II
PWY-7323	superpathway of GDP-mannose-derived O-antigen building blocks biosynthesis
COLANSYN-PWY	colanic acid building blocks biosynthesis

[1] Blue font indicates pathways identified with Lefse

[2] A negative score indicates negative association with infection. The relative abundance of the metabolic pathways is predicted to be lower in

PWY-1269	-0.5025
COLANSYN-PWY	-0.5024
NAGLIPASYN-PWY	-0.5018
PWY-6467	-0.5014
PWY-6892	-0.4999
PWY-5505	-0.4892
PWY-6895	-0.4869
PWY0-1586	-0.4827
PWY-6353	-0.4814
RIBOSYN2-PWY	-0.4801
PWY-7323	-0.4794
RHAMCAT-PWY	-0.4746
PWY-6147	-0.4718
GLUCARDEG-PWY	-0.4689
PWY0-1533	-0.4635
GALACTARDEG-PWY	-0.46
GLUCARGALACTSUPER-	-0.46
PWY-6969	-0.4596
FUC-RHAMCAT-PWY	-0.4578
PANTO-PWY	-0.4465
PWY-7539	-0.4435
TCA	-0.4433
P341-PWY	-0.4392
PWY-5104	-0.4372
PANTOSYN-PWY	-0.4329
CALVIN-PWY	-0.4328
PYRIDOXSYN-PWY	-0.4249
PWY0-845	-0.4189
ARG洂_xD800_倉SYN	-0.4183
PWY0-1261	-0.4167
FUCCAT-PWY	-0.4162
PWY-7111	-0.4105
NONOXIPENT-PWY	-0.4101
ILEUSYN-PWY	-0.4096
VALSYN-PWY	-0.4096

ARGORNPROST-PWY	-0.4058
HEXITOLDEGSUPER-PWY	-0.4049
HEME-BIOSYNTHESIS-II	-0.3986
PWY-7332	-0.3925
PWY-5101	-0.3919
GLYCOLYSIS	-0.3884
PWY-3001	-0.3852
PWY-7663	-0.3845
PWY-5345	-0.3827
PWY-5973	-0.3815
GLYCOCAT-PWY	-0.3804
PWY-1861	-0.3802
ANAEROFRUCAT-PWY	-0.3773
SER-GLYSYN-PWY	-0.3716
1CMET2-PWY	-0.3714
PWY-5103	-0.3706
RUMP-PWY	-0.3691
PWY-6737	-0.3689
PWY-3781	-0.3682
PWY-5918	-0.3656
HISTSYN-PWY	-0.3613
BRANCHED-CHAIN-AA-SYN-PWY	-0.3595
GLUTORN-PWY	-0.3566
ARGSYNBSUB-PWY	-0.3435
PWY-5484	-0.342
PWY-7199	-0.3416
PWY-7400	-0.3384
ARGSYN-PWY	-0.338
PWY-5677	-0.3324
TEICHOICACID-PWY	-0.3321
GLUCONEO-PWY	-0.3273
PWY-5705	-0.3263
P221-PWY	-0.3239
HSERMETANA-PWY	-0.3181

PWY-7090	-0.3168
PWY-6163	-0.315
POLYAMINSYN3-PWY	-0.3092
PWY490-3	-0.3089
PWY-5121	-0.3081
NONMEVIPP-PWY	-0.3056
PWY-7560	-0.3056
PWY0-1338	-0.3047
PYRIDNUCSAL-PWY	-0.3045
COMPLETE-ARO-PWY	-0.3021
ARO-PWY	-0.2997
GOLPDLCAT-PWY	-0.2994
REDCITCYC	-0.2993
PWY-7013	-0.2982
PWY-6897	-0.2911
TRNA-CHARGING-PWY	-0.2867
DTDPRHAMSYN-PWY	-0.282
ASPASN-PWY	-0.2769
THISYN-PWY	-0.2736
PWY-5347	-0.2685
PWY-7392	-0.265
OANTIGEN-PWY	-0.2635
DAPLYSINESYN-PWY	-0.2631
SULFATE-CYS-PWY	-0.2516
HOMOSER-METSYN-PWY	-0.2365
PWY-6891	-0.2318
MET-SAM-PWY	-0.2317
P164-PWY	-0.196
SO4ASSIM-PWY	-0.1947
HEMESYN2-PWY	-0.1916
PWY0-1241	-0.1911
PENTOSE-P-PWY	-0.1843
PWY-6263	-0.171

PWY-6901	-0.1704
P42-PWY	-0.1649
PWY-7374	-0.1641
PWY-7371	-0.164
CODH-PWY	-0.1626
POLYAMSYN-PWY	-0.1522
NAD-BIOSYNTHESIS-II	-0.1461
PWY-922	-0.142
PWY-7200	-0.1404
FASYN-ELONG-PWY	-0.1394
PWY-5910	-0.1373
PWY0-1061	-0.1287
PWY-5659	-0.1141
GLUCOSE1PMETAB-PWY	-0.1118
PWY-6383	-0.1118
PWY-7254	-0.1118
PWY1G-0	-0.1118
PWY-7196	-0.0825
FERMENTATION-PWY	-0.0641
GLYCOGENSYNTH-PWY	-0.0396
PWY-5913	-0.0288
PWY-6612	0.0037
GLCMANNANAUT-PWY	0.0154
FOLSYN-PWY	0.0169
PWY-5667	0.0174
PWY0-1319	0.0174
PWY-6700	0.0254
PWY-5100	0.0287
PWY-5695	0.034
PWY0-781	0.0464
PWY-6628	0.068
PWY-6630	0.068
ANAGLYCOLYSIS-PWY	0.089
P4-PWY	0.0909
COA-PWY	0.0982
PWY-6122	0.127
PWY-6277	0.127
PWY-2942	0.1334
PWY0-1296	0.1439

PWY-5097	0.1449
PWY-6121	0.1546
PWY-6471	0.1561
PWY-621	0.1589
PHOSLIPSYN-PWY	0.1643
PWY-7220	0.1657
PWY-7222	0.1657
PWY0-162	0.176
PWY-2941	0.1812
PWY-7219	0.1833
PWY-7234	0.1861
PWY4FS-7	0.1869
PWY4FS-8	0.1869
POLYISOPRENSYN-PWY	0.1912
DENOVOPURINE2-PWY	0.1926
PWY-6126	0.1953
LACTOSECAT-PWY	0.196
PWY-7197	0.196
PWY-5686	0.1988
PWY-841	0.1998
PWY-7208	0.2041
PWY-6125	0.2054
PWY-7229	0.2093
PWY-7187	0.2149
PWY-6609	0.2162
PWY-7228	0.2195
P122-PWY	0.2204
PWY-6386	0.2264
PWY-7184	0.2287
PWY0-166	0.2332
P124-PWY	0.2361
PWY-6387	0.2362
PWY-6385	0.2365
PEPTIDOGLYCANSYN-PWY	0.2379
PWY0-1298	0.2388
PWY-5384	0.2417
PWY0-1297	0.2506
PWY-6317	0.2759
PWY-6151	0.2809
PWY-6123	0.2863
PWY-5022	0.3036
PRPP-PWY	0.3056
PWY-7221	0.3306

UDPNAGSYN-PWY	0.3403
PWY-7211	0.3404
P161-PWY	0.3407
PWY-7377	0.3757
PWY-6470	0.3776
THRESYN-	0.4109
P441-PWY	0.4289
GLYCOLYSIS-E-D	0.435
P23-PWY	0.4743
PWY-5265	0.4743
PWY-4984	0.6248

Table S2. Weighted UniFrac distances between 16S sequences from synthetic bacterial population

**comparison**	**weighted UniFrac distance**
exp35-exp37	0.03
exp35-exp38	0.05
exp37-exp38	0.04
exp35-exp40	0.05
exp37-exp40	0.04
exp38-exp40	0.05
mean	*0.041*
SD	*0.008*
